# Environmental enrichment as an intervention to prevent behavioral and neurobiological consequences of maternal separation in rodents: a scope review

**DOI:** 10.1016/j.ynstr.2026.100800

**Published:** 2026-03-17

**Authors:** Natalia Ferreira de Sá, Nadyme Assad, Rosana Camarini, Deborah Suchecki

**Affiliations:** a Department of Psychobiology, Escola Paulista de Medicina, Universidade Federal de São Paulo, Brazil; b Department of Pharmacology, Instituto de Ciências Biomédicas, Universidade de São Paulo, Brazil

**Keywords:** Early life stress, Animal models, Psychiatric disorders, Neurodevelopment, Affective behaviors, Motivated behaviors

## Abstract

Neglectful care or disruption of the mother-infant relationship can alter the offspring's neurodevelopmental trajectory, inducing non-adaptative behaviors and negative neurobiological consequences. Environmental enrichment (EE), in turn, promotes the expression of animals' innate behaviors by providing cognitive, sensory, motor and social stimuli. Few studies have explored EE effects on animals with a previous history of early life stress, with maternal separation (MS) being the most frequently used paradigm. This scope review aimed to evaluate whether EE can prevent and/or reverse the deleterious effects induced by MS. Out of 182 retrieved papers, the 36 that were included assessed the outcomes of EE in maternally-separated animals. Behavioral endpoints included social, anxiety- and depression-like behaviors, memory and cognition, and alcohol intake. Neurobiological endpoints were HPA axis activity, neurogenesis, neuroplasticity, neuroinflammation, and resilience factors. We detected a great variation in MS and EE protocols, generating large variability of results. Nonetheless, robust evidence supports the beneficial EE effects in reversing or attenuating MS-induced learning and cognitive impairments. Moreover, EE shows promising effects in enhancing neurogenesis and the expression of resilience factors. Findings regarding affective and motivated behaviors are more variable, but they underline stronger benefits when EE is applied during the prepubertal and pubertal periods. The literature also suggests a sex dimorphism in EE responsiveness, with females being more resistant and requiring longer exposure to manifest the benefits. Overall, these findings reinforce the potential of EE as a non-pharmacological strategy to mitigate early life stress-induced alterations, while underscoring the need for standardized protocols and sex-specific investigations.

## 1 Introduction

### 1.1 Stress response in neonatal period and infancy

During infancy, in humans, rats and mice, the stress response mediated by the hypothalamic-pituitary-adrenal (HPA) axis differs from that of adolescent and adult individuals (Engel and Gunnar, 2020). Ontogenetic changes in the responsiveness of the adrenal glands to their trophic hormone, the adrenocorticotrophic hormone (ACTH), and the effectiveness of the corticosterone (in rats and mice)/cortisol (in primates) (CORT) negative feedback are the main explanations for this differentiated activity, known as the stress hyporesponsive period (SHRP) (Rosenfeld et al., 1991; Engel and Gunnar, 2020; Lupien et al., 2009; Sapolsky et al., 2000).

The SHRP spans from postnatal day (PND) 4 to 14 in rats (Witek-Janusek, 1988), and from PND 2 to 12 in mice (Enthoven et al., 2010). In humans, two-month old babies are able to mount a cortisol response to physical examinations, but the stress response diminishes by 3 months of age and several stressors fail to induce cortisol release until the first year of life. This physiologic failure of stress-induced cortisol secretion seems to result from a more functional cortisol negative feedback, and a decreased sensitivity of the adrenal to its trophic hormone, resembling the rodent SHRP (reviewed in Gunnar and Quevedo, 2007). Since high concentrations of CORT during this phase may be detrimental to proper brain development (Gunnar and Donzella, 2002; Schoenfeld et al., 1980), thus increasing susceptibility to psychiatric disorders (Faturi et al., 2010; Suchecki, 2018), the SHRP is regarded as a fundamental neurodevelopmental period that ensures adequate maturation of brain structures and functioning of the HPA axis, contributing to adaptive responses to stress throughout life (Wood and Walker, 2015).

Parental care is crucial for the SHRP. In humans, the timing of this period is not well established, although it is known to occur in early childhood (Gunnar and Donzella, 2002). During this developmental phase, maternal behaviors, such as breastfeeding, physical contact, smell and tone of voice, serve as fundamental regulators that program the infant's future behavioral and physiological stress responses (Császár-Nagy and Bókkon, 2018). In rodents, maternal behaviors regulate components of the stress response system: anogenital licking suppresses ACTH release, while feeding and suckling behaviors inhibit CORT production (Suchecki et al., 1993b; van Oers et al., 1998).

### 1.2 Animal models of neglect and abuse in childhood

During childhood, impoverished and unstable parental care, as well as neglect or abuse by primary caregivers have a major short- and long-term impacts on mental health, reflected by cognitive and emotional impairments (Duniec and Raz, 2011; Hofer, 1987; Teicher et al., 2003). Such adverse experiences can disrupt the maturation of brain circuits involved in stress response, and increase the vulnerability to depression, anxiety and posttraumatic stress disorder (Bick and Nelson, 2016; Callaghan and Tottenham, 2016; Chen and Baram, 2016; Hegde and Mitra, 2020; McLaughlin et al., 2019). Animal models of early adversity have revealed how elevated CORT concentrations during neurodevelopment trigger cascading effects throughout the lifespan. These include HPA axis dysregulation (Suchecki, 2018), neurobiological damage (Caldji et al., 1998; Francis et al., 2000; Jensen Peña and Champagne, 2013; Liu et al., 2000), and impaired expression of affective behavior (Faturi et al., 2010). The quality of maternal care appears to shape these outcomes through epigenetic mechanisms, with evidence suggesting that such programming can influence behavioral and neurobiological patterns across generations (Fish et al., 2004; Francis et al., 1999; Weaver et al., 2004).

In rodents, several protocols were developed to study the effects of absence or impaired maternal care. The maternal deprivation (MD) is a protocol based on disruption of the mother-infant relationship and consists of a single separation between mom and pups for 24 h. Several reviews describing the effects of MD on behaviors and neurobiological systems were published before (De la Fuente et al., 2009; Levine, 2001; Levine, 2005; Marco et al., 2009; Suchecki, 2018; Zanta et al., 2023; Ferreira de Sá et al., 2023) and we will refrain from describing them, since MD is not the focus of the present review.

Maternal separation (MS) is also a paradigm based on disruption of the mother-infant relationship, but, differently from MD, it consists of repeated daily periods of separation of the mother from the litter. In the literature, there is a significant diversity of this paradigm. There is no standard pattern regarding the choice of the litter's age, duration or frequency of the separation. The duration of separation usually varies between 3 and 6 h per day, starting on PNDs 1, 2 or 3 and lasting from 10 to 21 days. Some researchers keep the pups in the homecage during separation, maintaining their access to the nest and maternal olfactory cues, while removing only the mother to a new cage (Menezes et al., 2020; Réus et al., 2021; Wood and Walker, 2015, Workman et al., 2011), and some keep the mother in the homecage, moving the litter to another cage (Tiba et al., 2004, 2008; Catallani et al., 2008; Kawakami et al., 2013, 2016). Others place both mother and offspring in new cages (Hui et al., 2011; Berardo et al., 2016; Koe et al., 2016; Dandi et al., 2018), and in a few studies pups are separated not only from the mother, but also from their siblings (Doreste-Mendez et al., 2019; McKibben and Dwivedi, 2021a; Irie et al., 2023). An important remark is that most studies maintain the animals in a thermal blanket during the absence of the mother, given that the pups are unable to regulate their body temperature when away from the mother for long periods (Suchecki et al., 1993a). The methodological differences in these specific protocols lead to significant variability in the results obtained, especially when more parameters are added to the analysis, which will be discussed in more detail throughout this review.

Besides the disruption of the mother-infant relationship, the quality of maternal care is a relevant aspect. Pups raised by mothers displaying low frequency of licking and grooming behaviors and reduced arched-back nursing postures show higher concentrations of ACTH and CORT in response to acute stress than pups reared by highly attentive mothers (Liu et al., 1997). The limited bedding and nesting (LBN) material paradigm induces fragmentation of maternal care and is thus used to study the long-term effects of neglect and impoverished care (Walker et al., 2017). Evidence shows that this model enhances anxiety-like and fear behaviors (Guadagno et al., 2018) while impairing cognitive and social functions (Kohl et al., 2015; Naninck et al., 2015). Regarding physiological and neurobiological effects, this paradigm impairs hippocampal neurogenesis, neuronal migration and survival (Guadagno et al., 2018; Kohl et al., 2015; Naninck et al., 2015; Walker et al., 2017), and increases excitatory inputs and activity of neurons in the basolateral amygdala (BLA) (Guadagno et al., 2018). Interestingly, females appear to be more resilient to the effects of LBN than males (Walker et al., 2017).

### 1.3 Environmental enrichment

Given the detrimental effects of early adversity demonstrated in these animal models, researchers have explored interventions that might mitigate or reverse these outcomes. Environmental enrichment (EE) is a type of housing that facilitates the expression of the species' typical cognitive, sensory, motor and social behavioral repertoire (Hutchinson et al., 2005, 2005, 2005; Mo et al., 2016; Nithianantharajah and Hannan, 2006). Because enriched cages offer greater complexity and variety that is closer to the natural environment, it is easier to observe the animals expressing their innate behaviors (Hutchinson et al., 2005). Besides, EE improves life quality and promotes physical and psychological well-being of animals (Baumans and Van Loo, 2013).

There are two main pillars in EE protocols: the physical and the social enrichment. Regarding the physical features, enriched cages are usually larger than standard cages and may have multiple levels. They contain objects with different shapes, sizes and textures - such as plastic and wooden toys, platforms, tunnels, and running wheels - arranged in different combinations and positions, to promote the free exploration and manipulation of the environment. Social enrichment is also crucial, especially for social species, such as rodents. In this case, the animals are housed with conspecifics, and the number of animals per cage may vary, resulting in more or less complex social relationships.

EE protocols have been increasingly used in basic research to prevent, reduce or reverse the deleterious effects of chronic stress. Animals housed in enriched cages show less anxiety-like behavior (Chapillon et al., 1999; Friske and Gammie, 2005; Peña et al., 2006, 2009; Novaes et al., 2017), lower fear expression (Klein et al., 1994; Roy et al., 2001), faster habituation to novel places (Brenes et al., 2008; Zimmermann et al., 2001), less anhedonia and increased motivated behaviors (Brenes Sáenz et al., 2006), compared to animals in standard housing conditions. These behavioral outcomes are associated with increased neurogenesis, number of synapses and neuronal survival and proliferation, and greater complexity of neural circuits, which together, induce a more efficient stress processing and decreases non-adaptive behaviors (Simpson and Kelly, 2011; van Praag et al., 2000). Consequently, animals housed in enriched cages develop more effective coping strategies when facing stressful situations (Brenes et al., 2008, 2009), exhibit improved motor, cognitive, memory and learning skills (Greenough and Volkmar, 1973; Henderson, 1970; Kempermann et al., 1997; Rosenzweig and Bennett, 1972; Turner and Greenough, 1985), and present more variable behavioral and neurobiological responses than animals housed in standard cages (Kempermann, 2019; Simpson and Kelly, 2011).

In the last two decades there has been a significant increase in the number of publications involving EE due to its promising benefits and improved well-being of the animals. In contrast, studies using EE during or after disruption of the mother-infant relationship in protocols other than MS are limited. Another review of our research group already extensively discussed the application of EE after the MD paradigm (Ferreira de Sá et al., 2023). Regarding the LBN paradigm, only one study to our knowledge has examined EE effects, finding no reversal of spatial learning and memory impairments or depressive-like behavior, despite observing increased hippocampal long-term potentiation (LTP) (Cui et al., 2006). However, EE has shown promise in other maternal neglect protocols, such as reducing anxiety behaviors induced by early weaning in Balb/c mice (Iwata et al., 2007). Given that MS remains the most widely used approach in early life stress research, the present review focuses specifically on the potential effects of EE in mitigating outcomes from MS protocols.

The neonatal and childhood periods represent windows of vulnerability in which the brain is more sensitive to the action of stress (Andersen, 2003). We propose shifting this perspective to also consider this neurodevelopment period as a window of opportunity when positive interventions can be introduced to promote short- and long-term resilience. In this context, the review was elaborated to discuss the main findings of MS and EE neurobehavioural effects to contribute to the field and guide future research. Therefore, our main objective was to answer whether EE could prevent and/or reverse the deleterious effects induced by MS. To do so, we organized this review by presenting the behavioral and neurobiological effects of MS, followed by the impact of EE on these parameters.

## 2 Methods

### 2.1 Search strategies

Based on the PICO approach (Table 1), the following search strategy was adopted: ("environmental enrichment" OR "enriched environment" OR "complex housing") AND ("maternal separation" OR "maternal deprivation" OR "early life stress"). Even though MD and MS are different protocols, as discussed above, these terms are commonly used as synonyms in the literature. For this reason, we included the term “maternal deprivation” in the search strategy, in order to not miss any relevant data. After obtaining the results from the database PubMed, the titles and abstracts were read to select the papers that would be included in the present review for reading in full and data extraction.

**Table 1:** PICO methodology and search strategy.

Population	Rats and mice
**I**ntervention	Maternal separation and Environmental enrichment
**C**omparison	Animals not exposed to any intervention
**O**utcome	Anxiety-like behavior, depression-like behavior, social behavior, memory, ethanol intake, HPA axis, neurogenesis, and neuroinflammation

### 2.2 Inclusion and exclusion criteria

From the articles retrieved in the search, we included only basic research conducted in rats and mice, whose main purpose was to evaluate the effects of EE protocols used during or after MS. The EE protocols included were those that allow free exploration of the enriched cage and its objects, as well as those that provided voluntary exercise through a running wheel.

The outcomes should include, at least, one of the following behaviors: social, anxiety- and/or depressive-like behaviors, voluntary ethanol consumption, memory and cognition. We excluded from this review: studies conducted in other species, animal models of neurodegenerative and neurodevelopmental disorders, studies on prenatal stress, and those evaluating drugs of abuse other than alcohol.

### 2.3 Data extraction and analysis of variables

**Fig. 1:**
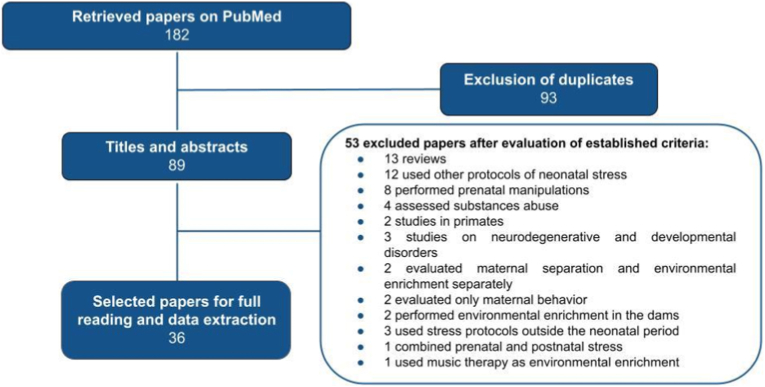
Flowchart outlining the steps of the methods used to search for and evaluate the studies included in this review, as well as the inclusion and exclusion criteria adopted.

A total of 36 papers, published between 2002 and 2024, fulfilled the inclusion criteria (Fig. 1). The information extracted from each paper were: 1) the duration and length of MS and EE; 2) type of MS and EE protocols; 3) age of the animals at EE; 4) age of the animals at testing; 5) behavioral tests and neurobiological analyses. As for the outcomes, the results of social and anxiety and depression-like behaviors, memory and cognition tests, alcohol consumption, HPA axis, neurogenesis and neuroplasticity, neuroinflammation and resilience factors were compiled and discussed. Methodological information is summarized in Table 2.

**Table 2:** Summary of the methodological information described in the studies included in this review.

Reference	Species and strain	Sex	MS protocol	Number of animals per cage	EE protocol			Timing of EE	Age at testing
					Toys, pipes, and platforms	Nesting material	Running wheels		
Francis et al. (2002)	Long-Evans rats	Not informed	3h/day, PND 1-14	8	Yes	No	No	PND 22-70, followed by SC	PND 110
Navailles et al. (2008)	Balb/c and C57Bl mice	Males and females	3h/day, PND 2-15	Not informed	Yes	Yes	No	PND 32-56	PNDs 56 and 70
Hui et al. (2011)	Sprague-Dawley rats	Males and females	3h/day, PND 2-14	10-12	Yes	No	Yes	PND 21-75	PND 61-75
Vivinetto et al. (2013)	Wistar rats	Not informed	4,5h/day, PND 1-21	7-10	Yes	No	Yes	PND 21-60, followed by SC	PND 67-74
Wang et al. (2014)	Sprague-Dawley rats	Not informed	3h/day, PND 1-10	Not informed	Yes	No	No	PND 21-77, 6h/day	PND 77
do Prado et al. (2016)	Sprague-Dawley rats	Males and females	4h/day, PND 2-20	3	Yes	Yes	No	PND 21-36	PND 36-56
Koe et al. (2016)	Wistar rats	Males	3h/day, PND 2-14	2-4	Yes	Yes	No	PND 56-70, followed by SC	PND 70-84
Berardo et al. (2016)	Wistar rats	Males and females	3h/day, PND 2-21	4	Yes	No	Yes	PND 21-42, followed by SC	PND 42-72
Dandi et al. (2018)	Wistar rats	Not informed	3h/day, PND 1-21	5-7	Yes	No	Yes	PND 23-65, followed by SC	PND 67-76
González-Pardo et al., 2019a	Wistar rats	Males	4h/day, PND 1-21	10	Yes	No	Yes	PND 21-65, followed by SC	PND 90-97
González-Pardo et al. (2019b)	Wistar rats	Males	4h/day, PND 2-21	10	Yes	Yes	Yes	PND 21-60, followed by SC	PND 90
Doreste-Mendez et al. (2019)	Sprague-Dawley rats	Males and females	3h/day, PND 1-21	4	Yes	Yes	No	PND 22-77 or PND 78-134	PND 78-80 or 135-137
Odeon and Acosta (2019)	Wistar rats	Males	1h/day, PND 2-20 Exposed to cold (4 °C)	6	Yes	No	Yes	PND 29-59, preceded (PND 22-28) and followed (PND 59-66) by voluntary ethanol intake in SC	PND 66
Menezes et al. (2020)	Wistar rats	Males	3h/day, PND 1-10	6-10	Yes	No	No	PND 21-80, followed by SC	PND 81-97
Cordier et al., 2020	Wistar rats	Males	4,5h/day, PND 1-21	8-10	Yes	No	Yes	PND 21-62	PND 61-63
Mohtashami Borzadaran et al. (2020)	Wistar rats	Males	3h/day, PND 1-21	Not informed	Yes	No	Yes	PND 22-60, followed by SC	PND 60
Hegde et al. (2020)	Wistar rats	Males	3h/day, PND 2-14	Dam and pups	Yes	Yes	No	PND 2-21, followed by SC	PND 60-70
Joushi et al. (2021)	Wistar rats	Males and females	3h/day, PND 1-21	6-9	Yes	Yes	Yes	PND 22-34, followed by SC	PND 35-42
Borba et al. (2021)	Wistar rats	Males and females	3h/day, PND 1-10	Not informed	Yes	No	Yes	PND 21-31, or 21-41, or 21-61, during 3h/day	PND 31, 41 or 61
Mansouri et al. (2021)	Wistar rats	Males	3h/day, PND 1-14	8-9	Yes	No	No	After weaning	PND 42-50
Réus et al. (2021)	Wistar rats	Males and females	3h/day, PND 1-10	Not informed	Yes	No	Yes	PND 21-31, or 21-41, or 21-61, during 3h/day	PND 21, 31 or 41
Huang et al. (2021)	C57BL/6J mice	Males	4h/day, PND 2-21	4-5	Yes	No	Yes	PND 22-56 or PND 57-91	PND 92-97
McKibben and Dwivedi, 2021b	Holtzman rats	Males and females	3h/day, PND 1-14	Not informed	Yes	Yes	No	PND 21-90	PND 81-95
McKibben and Dwivedi, 2021b	Holtzman rats	Males and females	3h/day, individually PND 1-14	Not informed	Yes	Yes	No	PND 21-90	PND 80-90
Wei et al. (2021a)	Balb/c mice	Males	4h/day, PND 3-21	6	Yes	No	No	PND 22-110	PND 60-90
Wei et al. (2021b)	Balb/c mice	Females	4h/day, PND 3-21	9-10	Yes	No	No	PND 22-110	PND 90-95
Wei et al., 2022	Balb/c mice	Females	4h/day, PND 3-21	6	Yes	No	No	PND 22-110	PND 80-102
Joushi et al. (2022)	Wistar rats	Males and females	3h/day, PND 1-21	6-9	Yes	Yes	Yes	PND 22-34, followed by SC	PND 30-40
Ji and Xia, 2022	C57BL/6J mice	Males	6h/day, PND 1-21	3-5	Yes	No	Yes	PND 1-40	PND 40 and PND 60 (behavior analysis) and PND 50 (neuroinflammation)
Ji et al., 2022a	C57BL/6J mice	Males and females	6h/day, PND 1-21	Not informed	Yes	No	Yes	PND 21-41 or 21-61	PNDs 41 and 61
Ji et al., 2022b	C57BL/6J mice	Males	6h/day, PND 1-21	Not informed	Yes	No	Yes	PND 1 to 4 weeks; 5 to 8 weeks; 9 to 12 weeks	PND 91
Wei et al., 2023	Balb/c mice	Females	4h/day, PND 3-21	4-5	Yes	No	No	PND 22-110	Noi informed
Wei et al., 2023	Balb/c mice	Males	4h/day, PND 3-21	6	Yes	No	No	PND 22-110	PND 52-75
Cevik et al., 2023	Wistar rats	Not informed	3h/day, PND 1-21	Not informed	Yes	No	Yes	PND 21-51	PND 51-59
Joushi et al. (2024)	Wistar rats	Males	3h/day, PND 1-21	6-9	Yes	Yes	Yes	PND 22-34 + Intranasal oxytocin administration	PND 30-34
Irie et al., 2023	Sprague-Dawley rats	Males	3h twice daily, individually, PND 2-20	6	Yes	Yes	Yes	PND 21-35	PND 35

IN: intranasal; PND: postnatal day; SC: standard cage.

## 3 Results

### 3.1 Memory and cognition

**Fig. 2:**
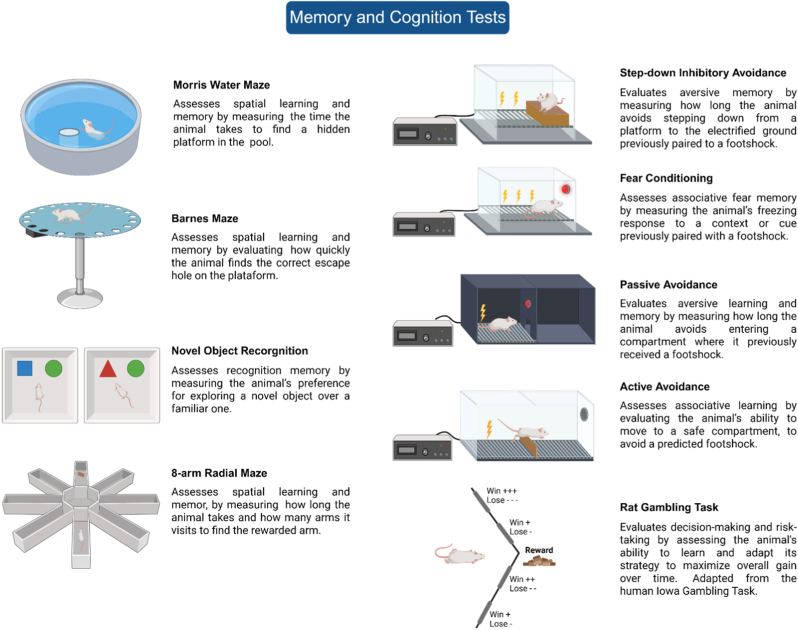
The main tests used to study different types of memory and decision-making. Figure created in BioRender.com.

In general, the data show that MS impairs the performance of animals in several memory and cognitive tasks, including those related to spatial memory (Aisa et al., 2007), novel object recognition (Aisa et al., 2007; Marco et al., 2009) and aversive memory (Guijarro et al., 2007). Fig. 2 presents the main tests used in the studies included in this session. This early life adversity also decreases neurogenesis in the CA1, CA3 and dentate gyrus (DG) of the hippocampus (Aisa et al., 2009; Huot et al., 2002). However, there is also evidence of MS-induced improvement in these tasks (Pryce et al., 2003; Schäble et al., 2007; Weiss et al., 2001) and the source of discrepancy is related to the age when MS is performed, to the sex and strain of the animals.

Most studies report that MS impairs spatial memory, tested in the Morris water maze (MWM) and in the Barnes maze, and this deficit is reversed by EE (Dandi et al., 2018; Hui et al., 2011; Joushi et al., 2021; Menezes et al., 2020; Cordier et al., 2021; Wang et al., 2014). Moreover, MS reduces LTP, a form of synaptic plasticity with an important role in memory and learning, which is reversed by EE (Joushi et al., 2021). Only one paper reported improved performance in the MWM after MS, and although EE enhanced the performance of the control (CTL) group, it had no effect on MS-exposed rats (Cevik et al., 2023).

Furthermore, male rats exposed to MS between PNDs 1 and 10 exhibit a compromised cognitive flexibility in the MWM than CTL animals. Eight weeks of EE, from weaning until testing, reverse this impairment (Menezes et al., 2020). Interestingly, in the Barnes maze, another spatial memory task, CTL animals adopt random spatial strategies, while their EE counterparts use direct and systematic strategies, suggesting greater neural complexity (Cordier et al., 2021). Moreover, 4 daily hours of MS from PND 2 to 20 impaired spatial memory in the 8-arm radial maze, during a win-shift reward task, with a reversal by EE in male and female Sprague-Dawley rats (do Prado et al., 2016). In the rat gambling test, an adaptation of the Iowa test for humans, 3 daily hours of MS for the first 3 weeks of life impaired decision-making by decreasing the percentage of advantageous choices, i.e., choices that led to less food pellets, but also, to shorter time-outs for wrong choices, when animals could not get the reward. Like in the previous studies, this impairment was reversed by EE (Joushi et al., 2024).

Although the effects of MS on spatial memory are not unanimous, those induced by EE are quite consistent, with improved performance of both CTL and MS animals (González-Pardo et al., 2019a). However, these results seem to be sex- and age-dependent. For instance, PND 31 and PND 41 male and female rats, separated from their mothers for 3 h between PNDs 1 and 10 and exposed to EE for 10 or 20 days after weaning, explored less the open field (OF) 24 h after a first exposure to the apparatus, e.g., they were able to retain the habituation memory. Nonetheless, at PND 61 (40 days in EE), this ability was impaired by MS and reversed by EE (Réus et al., 2015). This finding indicates that MS effects on this type of memory require time to manifest and that prolonged EE is effective in rescuing this ability.

Regarding the NOR test, discrepant effects induced by MS have been reported. Males and females subjected to MS show impairments in this test and EE reverses these effects in short-term memory (STM), 1 h (Wei, Li, et al., 2021; Wei, Zhang, et al., 2021), 3 h (Menezes et al., 2020), and 4 h (Wang et al., 2014) after training trial, and in long-term memory (LTM), 24 h after training trial (Menezes et al., 2020). On the other hand, some studies report no effects of MS or EE in this test (Dandi et al., 2018; Joushi et al., 2021; Vivinetto et al., 2013). The divergence can be explained by methodological differences, such as the animal strain and the length of MS and EE. Studies failing to detect EE effects used Wistar rats subjected to prolonged MS (PNDs 1-21) combined with shorter EE exposure periods (14-40 days) (Dandi et al., 2018; Joushi et al., 2021; Vivinetto et al., 2013). In contrast, studies demonstrating beneficial effects of EE following MS used mice exposed to shorter MS periods (PNDs 3-21 or PNDs 1-10) combined with longer EE duration of at least 2 months (Menezes et al., 2020; Wei, Li, et al., 2021; Wei, Zhang, et al., 2021).

The effects of MS on emotional memory are not consensual throughout the studies, with some showing a small effect in male (Guijarro et al., 2007) or no effect in female rats (Sun et al., 2014), whereas others report increased freezing behavior in fear conditioning in male (Toda et al., 2014) and enhanced spontaneous recovery of extinguished fear in female rats (Xiong et al., 2014). Regarding the studies included in this review, the harmful effect of MS in the step-down inhibitory avoidance (IA) was observed when rats were tested 1 h (Vivinetto et al., 2013) or 24 h after the training session (Menezes et al., 2020), indicating a non-adaptative deficit in aversive memory, and in both cases, EE was able to reverse this effect (Vivinetto et al., 2013; Menezes et al., 2020). However, no differences in the active avoidance task were observed between CTL and MS male and female rats separated between PNDs 1 and 14; males showed longer scape latency than females and EE reduced scape latency in males (McKibben and Dwivedi, 2021a). In a subsequent study the same group showed no effect of MS in the performance in active avoidance, although females had shorter scape latency than males, but repeated exposure to 2 h/day of restraint stress increased the escape latency of females to the same level of males, without any EE effect (McKibben and Dwivedi, 2021b). In mice, MS effects on avoidance tests appear to depend on whether it is passive or active, and on the sex of the animal. In the passive avoidance test, MS females show short latency to enter the dark compartment paired with a footshock, with a reversing effect by EE (Wei et al. 2023). However, in the step-on test, in which the animals must step on a platform to avoid a footshock, MS increased the latency, and EE had no effect (Wei et al., 2023). In male mice, MS animals showed impaired performance in the passive avoidance and EE could not rescue the behavior 24 h or one week after training. Interestingly, latency of MS mice increased one week after training and EE *per se* increased the latency to enter the aversive chamber. Immediately and one week after training, MS impaired the response in the step-on test and EE partially rescued the behavior to the level of non-separated counterparts (Wei et al., 2023).

Overall, the data reviewed in this session underscore that MS and EE, whether associated or not, affects memory and learning in a task-, memory type- and sex-dependent fashion. Therefore, neither manipulation has generalizable effects, although EE effects are more consistent than those induced by MS. It is worth mentioning that different types of memory recruit distinct brain structures and involve different neurobiological processes, suggesting that both the MS and EE act in different ways in each situation. However, in general, EE shows consistent and promising results reversing the deleterious effects of MS on memory deficits.

### 3.2 Affective behaviors

**Fig. 3:**
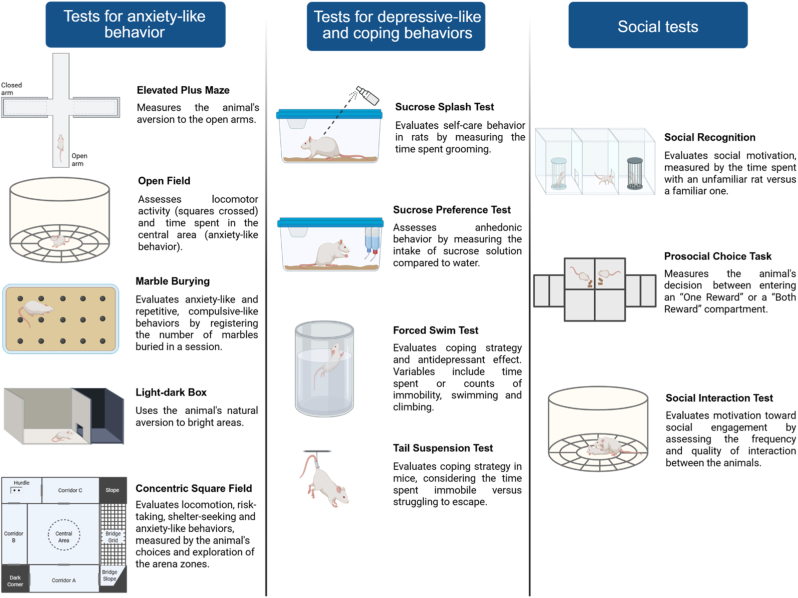
Tests employed to assess anxiety-like, depression-like, social and coping behaviors. Figure created in BioRender.com.

Fig. 3 presents the behavioral tests used to assess anxiety, depressive-like and social behaviors.

#### 3.2.1 Anxiety-like behavior

MS induces anxiety-like behavior during adolescence, with decreased number of entries in the open arms and more time spent in the closed arms of the elevated plus maze (EPM) (Shin et al., 2016; Daniels et al., 2004; Qin et al., 2021). There are also reports of diminished time spent in the center of the OF (Qin et al., 2021; Malcon et al., 2020) and in the light chamber of the light-dark box (Malcon et al., 2020).

Among the studies included in this review, we found a great diversity of results, especially in those using rats. Some of them show that neither MS nor EE affected ambulation and exploration in the OF (Borba et al., 2021; Koe et al., 2016; Menezes et al., 2020), whereas others report that MS reduces exploration and time spent in the central area of the OF, an effect that is reversed (Francis et al., 2002; Hegde et al., 2020; Ji and Xia, 2022a; Joushi et al., 2021; Joushi et al., 2021) or prevented by EE (Ji and Xia, 2022). Still, in one study no differences in locomotor activity was observed between CTL and MS animals, but EE increased the frequency of rearing in both groups (Dandi et al., 2018).

EE decreases exploration in the OF in male rats (Cevik et al., 2023; Mansouri et al., 2021, 2021, 2021; Wei et al., 2022, 2022, 2022) and in female mice (Mansouri et al., 2021; Wei et al., 2022). In addition, there is an age-dependent effect of EE, insofar it reduces exploration in adolescent rats and increases the time spent in the center in adults (Doreste-Mendez et al., 2019). Reduced exploration of new environments (Mansouri et al., 2021) by animals housed in enriched cages can be explained by the frequent changing of objects and of their placement in the home cages leading to faster habituation to novelty. Consequently, the interest and motivation of the animals to explore tends to be lower compared to animals housed in standard cages, where the environment is poor in stimuli. Animals living in enriched environments also seem to process new information faster than those housed in conventional cages. So, the decreased exploration does not necessarily indicate increased anxiety-related behavior. Rather, it may reflect the animals’ greater capacity of habituation (van der Veen et al., 2015).

However, these effects are not unanimous. Regardless of the lack of MS effect on exploratory and anxiety-like behaviors in some studies, EE increases total ambulation in the EPM (McKibben and Dwivedi, 2021a) and in the elevated zero maze (González-Pardo et al., 2019a). Still in the EPM, evidence also shows that MS decreases the time spent and the number of entries into the open arms (Dandi et al., 2018; Koe et al., 2016), increases the frequency of risk-taking behavior (Dandi et al., 2018) and decreases risk assessment (Koe et al., 2016) in male Wistar rats, and these impairments are all reversed by EE (Dandi et al., 2018; Koe et al., 2016). However, some evidence shows that MS increases the time spent in the closed arms, and neither 21 (Mansouri et al., 2021) nor 30 days (Cevik et al., 2023) of EE reverse this effect in male Wistar rats and 88 days of EE also do not reverse this prejudice in female Balb/c mice (Wei et al., 2022).

The results can also differ depending on the age when the behavioral test is performed, the sex of the animals, the duration of EE protocol, and the interaction among these variables. Some studies report a sex effect on the EPM measures (Menezes et al., 2020), with females showing less anxiety-like behavior than males (McKibben and Dwivedi, 2021a). The length of EE protocol and, consequently, the age of the animals when they are tested, influence the results. For instance, 10 days of EE starting on PND 21, increases exploration of male Wistar rats in the open arms of the EPM (PND 31), but in females, this effect is observed only after 40 days of EE (PND 61). The MS effect of increasing the anxiety index in both sexes is observed only on PND 41, and 20 days of EE, reverse this change, but only in female rats (Réus et al., 2021). Similarly, 20 days of EE is sufficient to increase the distance traveled and the time spent in the central area of the OF in male C57BL/6J mice tested on PND 41, but in females this same result is observed only after 40 days of EE, when they are tested on PND 61 (Ji et al., 2022b). Thus, females appear to be more resistant than males to the effects of EE.

In addition to sex differences, the developmental timing of EE is crucial for its effects on anxiety-like behaviors, with earlier interventions proving more beneficial. C57BL/6J mice exposed to EE during prepubertal or pubertal phases following MS show reduced anxiety-like behaviors in adulthood (increased time in the central area of the OF and time in open arms in the EPM), effects not observed when EE is applied during adulthood (Ji et al., 2022b). Similarly, EE during juvenility and adolescence more effectively reduces MS-induced anxiety than adult EE interventions (Huang et al., 2021), confirming that early EE application yields superior outcomes.

Another important aspect in the analysis of EE effectiveness is related to the test used to assess anxiety-like behavior. We observed that the anxiolytic effect of EE in MS-exposed animals is more robust in the home cage emergence test, which measures the latency for an animal to leave its home-cage and cross into an adjacent one (Koe et al., 2016). In the novelty suppressed feeding, MS increases the latency to initiate the intake of chow (Francis et al., 2002) and in the marble burying test MS produces more stereotyped behaviors, increases the number of marbles buried and self-grooming in rats (Mansouri et al., 2021) and mice (Wei, Li, et al., 2021; Mansouri et al., 2021; Wei et al., 2022), with EE reversing all the behavioral changes.

#### 3.2.2 Depressive-like and coping behaviors

Anhedonia and avolition are relevant behaviors for the study of depression and are commonly expressed in animals exposed to MS, consisting on reduced self-grooming behavior in the sucrose splash test (Nouri et al., 2020; Réus et al., 2015) and the lesser preference for consuming sucrose solution (Hui et al., 2011). MS consistently induces lower preference for sucrose, which is reversed (Hui et al., 2011; Ji and Xia, 2022; McKibben and Dwivedi, 2021a, 2021b) and prevented (Ji and Xia, 2022) by EE, demonstrating its potential to increase motivated behaviors and self-care in animals.

Similar to the previous behavior, EE benefits are dependent on the developmental stage when it is applied, and early EE is more effective to rescue sucrose preference in MS-exposed animals than in adulthood (Huang et al., 2021; Ji and Xia, 2022). Besides, sex is also a major variable in depressive-like behavior. Again, a shorter period of EE in males increases sucrose preference in male mice, but in females this effect is observed after a longer EE (Ji and Xia, 2022).

The forced swim test (FST) is widely used to assess depressive-like behavior, although its validity as a test to evaluate relevant behaviors for depression is controversial (Barroca et al., 2022; Molendijk and de Kloet, 2019, 2015). In most studies selected, MS had no effect on immobility time in the FST (Cordier et al., 2021; Doreste-Mendez et al., 2019; González-Pardo et al., 2019a; McKibben and Dwivedi, 2021a). There is evidence showing that MS reduces immobility during the test in a sex-dependent way (Wei et al., 2022), with males displaying more climbing behavior than females (Ji and Xia, 2022; McKibben and Dwivedi, 2021a, 2021b). The effects of EE are also controversial. While some studies show an increase in immobility time in animals exposed to EE in adulthood, but not in adolescence (Doreste-Mendez et al., 2019), and a decrease in climbing behavior (McKibben and Dwivedi, 2021a), others report a decrease of immobility in mice, especially in males (Ji and Xia, 2022), and an increase of swimming in rats, which the authors interpreted as enhanced impulsive behaviors induced by EE (González-Pardo et al., 2019a) or use of active coping strategy. Other studies show that MS increases immobility time in the test, which is reversed in male and female Wistar rats after 40 days and after 20 days of EE, respectively (Borba et al., 2021), the opposite sexual effect of what was observed for anxious-type behavior. This reversal is more significant when EE is performed in the juvenile period or adolescence, since in adulthood there is no effect (Ji and Xia, 2022) or the effect is less expressive (Huang et al., 2021). Besides, it is important to note that these reversing effects are not observed in Sprague-Dawley rats (Hui et al., 2011), suggesting that, not only sex and developmental stage, but also the strain of the animals are important variables to be considered.

The conflicting results obtained with the FST may derive from the fact that this test relies on active and passive coping strategies and not necessarily depressive-like behaviors. These coping strategies are directly related to the individual variability, including genetic variations, behavioral patterns and neurochemical factors (Armario, 2021; Commons et al., 2017). Antidepressant drugs increase active behaviors (swimming and climbing) in the FST, so the test is useful for pharmacological validation (Piras et al., 2010; Porsolt et al., 1978), but its use for assessing depressive behaviors is debatable. Another test to assess coping strategies in the face of stress is the tail suspension test. To the best of our knowledge, only one study used this test to assess EE potential to reverse MS deficits in female mice, showing no effect of MS on, and an increase in immobility induced by EE (Wei et al., 2022), indicating that these animals prefer to resort to passive coping strategies.

### 3.3 Motivated behaviors

#### 3.3.1 Social behavior

Rats and mice are social animals, therefore, reduced interest and motivation to explore a conspecific may indicate impairment of this innate behavior, as well as an increase in behaviors relevant to the study of psychiatric disorders in animal models, such as social anxiety, depression, post-traumatic stress disorder, and schizophrenia (Sandi and Haller, 2015), since these social impairments are frequently observed in patients with these emotional disorders (APA, 2022). Animals subjected to MS express less social interaction and more aggressive behaviors (Haller et al., 2014; Kompier et al., 2019), in addition to impairments in social recognition and discrimination tasks (Lukas et al., 2011; Ognibene et al., 2008).

Among the studies that evaluated social discrimination, most failed to show any MS effect on social recognition tasks or on female preference for males (Joushi et al., 2021; Wei, Zhang, et al., 2021; Wei, Li, et al., 2021). In some cases, EE improved social recognition and discrimination in rats (Joushi et al., 2021) and mice (Wei, Li, et al., 2021), despite the absence of MS effect on this ability.

Regarding social motivation, MS reduces social interest for unfamiliar conspecifics and EE reverses this impairment in both male and female prepubertal rats (Joushi et al., 2021) and adult mice (Wei, Li, et al., 2021; Wei, Zhang, et al., 2021). Moreover, this EE effect can be observed either by itself (Joushi et al., 2021; Wei, Li, et al., 2021; Wei, Zhang, et al., 2021) or in association with oxytocin (OT), with a synergistic effect of both treatments (Joushi et al., 2022). MS-induced changes in social behavior appear to be accompanied by changes in the oxytocinergic system, including less oxytocinergic neurons in the magnocellular region of the paraventricular nucleus of the hypothalamus (PVN), augmented neurogenesis of these neurons and increased expression of oxytocin receptors (OTR) in the BLA in male and female mice (Wei, Li, et al., 2021; Wei, Zhang, et al., 2021). EE attenuates these effects by increasing oxytocinergic neurons in the parvocellular region of the PVN, enhancing neurogenesis in the prelimbic cortex, and increasing the number of functional synapses in both the BLA and the prelimbic cortex, thereby improving social and cognitive processing (Wei, Li, et al., 2021; Wei, Zhang, et al., 2021). The oxytocinergic system plays an important role mediating EE outcomes. Administration of an OT antagonist reduces the protective effects of EE on social and depressive-like behaviors after chronic stress protocols (Bashiri et al., 2021). In addition to EE, intranasal administration of OT improves the performance of MS animals in the rat gambling test, enabling them to make better decisions (Joushi et al., 2024), showing that this system is related to behaviors beyond social behavior, playing an important role in other cognitive functions.

Prosocial behavior is also affected by MS and EE protocols and by the oxytocinergic system. Male and female Wistar rats subjected to MS express less prosocial behavior in a prosocial choice test, and this deficit is reversed by EE and by OT administration (Joushi et al., 2022). Furthermore, there is a sex-dependent effect of MS and EE and an influence of the phase of the estrous cycle on the expression of social behaviors. MS decreases social interest in females during metestrus and diestrus phases, and EE increases sociability during metestrus (Wei, Zhang, et al., 2021).

Although EE is known to increase motivation and social preference (Garbugino et al., 2016; Rae et al., 2024), one study showed that in addition to MS, EE by itself, decreased social motivation, reflected by greater latency to explore an unknown conspecific in the three-chamber test; however, the association between EE and OT was capable to reverse this deficit in social behavior (Mansouri et al., 2021). EE protocols may adopt not only physical, but also social enrichment, with a large number of animals per cage. In the abovementioned study, 8 to 9 animals were housed together in the same cage. Thus, it is possible that the great amount of social stimuli reduced the animals' motivation to explore and interact with unknown conspecifics. Conversely, EE with high population density may add new challenges to the environment due the greater social complexity, establishing a hierarchical configuration, with relationships of domination and subordination (Barker et al., 2017), which can influence social behaviors.

It is important to discuss physical enrichment and social enrichment, and differentiate these two protocols. Social enrichment by itself is sufficient to increase open-arm exploration in the EPM, compared to individually housed rats. However, when associated with physical enrichment it also reduces aggressive behaviors and depressive-like responses (Workman et al., 2011). Therefore, the choice of EE protocol should be well-thought, because each pillar of EE (social and physical enrichment) leads to different outcomes for social, affective and motivated behaviors.

#### 3.3.2 Voluntary ethanol consumption

MS increases ethanol intake and preference, both in adolescence and adulthood (Cruz et al., 2008; Daoura et al., 2011; Huot et al., 2001; Leichtweis et al., 2020). To date, few studies have sought to elucidate the effects of EE during or after protocols of disrupted mother-pup relationship on alcohol intake and the results are controversial. In one study, even though there was no MS effect on ethanol preference, EE for 21 days increased this parameter only in male rats, in adolescence and adulthood. In females, EE increased the time spent on the light side of the light–dark box and the number of transitions between the two compartments. In males, EE increased rearing and the time spent in the open arms of the EPM, in addition to enhancing exploratory and risk-taking behaviors in the concentric square field (Berardo et al., 2016). Thus, EE appears to reduce the expression of anxiety-like behaviors across different tests, although the authors discuss that greater risk-taking behavior observed in EE animals could be associated with increased impulsivity, likely related to their higher alcohol intake.

In contrast, another study showed that 30 days of EE were sufficient to reverse the increase in voluntary ethanol intake in MS males. Furthermore, MS-exposed rats also showed greater exploration of aversive areas in the OF, EPM, and light-dark box (Odeon and Acosta, 2019). However, this exploration was interpreted by the authors as excessive, possibly indicating a non-adaptative behavioral pattern, since the most adaptive response is the one that balances the animal's safety and the natural expression of exploratory and curious behavior. Therefore, this result was interpreted by the authors as increased impulsive behavior, which was correlated with higher ethanol intake. Regarding the EE effect, an anxiolytic effect was observed in CTL animals, whereas in MS animals the reduction of the apparatus over-exploration was interpreted by the authors as a decrease in impulsive behavior (Odeon and Acosta, 2019). However, it is important to highlight that in this study, although the MS protocol lasted only 1 h per day, between PNDs 2 and 20, it was combined with another stressor, cold exposure (4 °C). Thus, the results regarding the EE effects on ethanol intake in neonatally stressed animals are conflicting, mainly due to the very limited number of studies on the topic and the lack of homogeneity and comparability between the available findings.

### 3.4 Neuroinflammation and pain

**Fig. 4:**
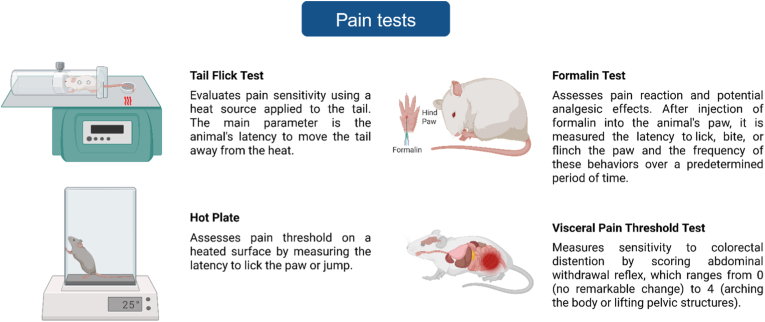
Behavioral tests employed to assess pain sensitivity. Figure created in BioRender.com.

Neuroinflammation and oxidative stress are associated with chronic pain, and highly correlated with the development of depression in humans (Czarny et al., 2018). In rodents, adversities in infancy affect the perception of pain. From the perspective of psychological stress and neuroinflammation, chronic pain can culminate in depression- and anxiety-like behaviors (Kimura et al., 2022). Many studies report that MS induces greater pain sensitivity, with increased nociceptive response and emotional pain perception, decreased pain thresholds, and prolonged pain response (Amini-Khoei et al., 2017; Paniagua et al., 2020; Salberg et al., 2020; Uhelski and Fuchs, 2010; Vilela et al., 2017, 2021). However, this susceptibility seems to depend on the nature of the stimulus (thermal, chemical or neuropathic pain, Fig. 4) (Genty et al., 2018; Butkevich et al., 2016). On the other hand, some studies report greater resistance to acute pain in animals submitted to MS, with decreased sensitivity and increased pain response threshold (Chen and Jackson, 2016; Ströher et al., 2019; Weaver et al., 2007). EE, in turn, attenuates various types of chronic pain, reducing inflammation and its comorbidities, such as anxious- and depressive-type behaviors in animal models (Kimura et al., 2022).

A small number of studies aimed to assess how EE affects MS consequences on systems related to pain sensation and perception. No influence of MS or EE was observed on the animals' response in the tail flick test (Menezes et al., 2020; Mohtashami Borzadaran et al., 2020). However, in the hot plate and formalin tests, MS animals exhibited, respectively, shorter latency to lick the paw and jump and higher score in the pain scale, evidencing greater sensitivity and vulnerability to pain, which were reduced by EE (Mohtashami Borzadaran et al., 2020). The lack of effect in the tail flick test can be explained by the aforementioned differences in pain perception to different types of stimuli. However, in general, MS augments sensitivity to pain and EE appears to be a useful tool to mediate and attenuate this maladaptive response.

Furthermore, studies that measure visceral pain threshold, reveal the importance of sex and the neurodevelopment period when EE is performed. MS increases pain sensitivity and EE protocols in prepubertal and pubertal phases reverse this effect in male mice, even when the animals are tested 5 weeks after the end of the protocol, but this beneficial effect is not observed when EE is performed in adulthood (Ji et al., 2022a, 2022b). Besides, when performed from the neonatal period until the pubertal phase, EE prevents MS-induced pain hypersensitivity, increasing the pain threshold immediately after the end of EE, and 20 days after housing in a standard cage (Ji and Xia, 2022). Regarding sexual differences, male mice required a shorter period of EE than females to increase the animals' pain threshold (Ji et al., 2022a).

Chronic stress in childhood or adulthood has a strong effect on neuroinflammatory processes, leading to increased pro-inflammatory factors that contribute to negative behavioral consequences, such as increased expression of anxious and depressive-type behaviors (Andersen, 2022; Brydges and Reddaway, 2020; Calcia et al., 2016; Dutcher et al., 2020; Munhoz et al., 2008) and greater predisposition to neurodegeneration (Desplats et al., 2020). EE, in turn, appears to protect the immune system from changes resulting from early stress, representing a possible target for prevention and resilience to psychiatric illnesses (Reddaway and Brydges, 2020).

MS induces hippocampal neuroinflammation, reflected by increased levels of IL-1 and IL-6 (Roque et al., 2016), and decreases neurogenesis in this brain structure (Nicolas et al., 2022). MS also induces hypothalamic neuroinflammation through activation of microglia, with increased expression of TNF-α (Roque et al., 2016), and reduces the concentration of anti-inflammatory cytokines, such as IL-10 (Dimatelis et al., 2012). Evidence from the studies included in the present review indicates that EE has a potential preventive effect against MS-induced neuroinflammation. When performed between PNDs 1 and 40, EE prevents the increase of the pro-inflammatory cytokines TNF-α and IL-1β, as well as the decrease of the anti-inflammatory cytokine IL-10 in the medial prefrontal cortex, BLA and PVN in animals subjected to 6 h of MS for 21 days (Ji and Xia, 2022). However, another study reported that MS had no effect on NADPH oxidase 2 and anti-inflammatory cytokines IL-4 and IL-10, but enhanced the peripheral concentrations of TNF-α, a pro-inflammatory factor, and this effect was no longer observed after 2 weeks of EE (do Prado et al., 2016). Furthermore, MS increases oxidative stress, but distinctly between sexes. In males, the concentrations of carbonylated proteins, reactive species to thiobarbituric acid, and nitrite/nitrate, are high on PNDs 31, 41, and 61 post-MS. In females, this damage is only observed at PND 61 post-MS. In addition, MS reduces antioxidant factors, such as superoxide dismutase, catalase, and sulfhydryl content. EE for 10, 20, or 40 days rescues these oxidative stress indices to CTL levels in males, but has no effects in females, whereas reduced antioxidants are reversed by 10 days of EE in females and 20 days in males (Réus et al., 2015).

### 3.5 HPA axis response

In rats and mice, the neonatal HPA axis stress response is regulated by maternal presence (Faturi et al., 2010) and specific maternal behaviors (Suchecki et al., 1993b; Van Oers et al., 1998). Therefore, disruption of the mother-offspring relationship results in immediate and long-term dysregulation of the HPA axis activity in rodents. In male rats, MS leads to enhanced CORT response to a brief handling in adulthood (Kalinichev et al., 2002), impaired CORT negative feedback mechanism, measured in the dexamethasone suppression test, due to, respectively, increased gene expression of mineralocorticoid receptor (MR) in hippocampal subfields and reduced hippocampal and cortical glucocorticoid receptors (GR) gene expression (Ladd et al., 2004). MS induces higher basal ACTH levels (Daniels et al., 2004), increased corticotropin-releasing factor (CRH) concentration in the PVN (Aisa et al., 2007, 2008; Plotsky and Meaney, 1993) and in the median eminence (Ladd et al., 1996).

Regarding CORT concentrations, some studies included in this review indicate that there is only a sex difference in basal plasma levels, with no influence of MS or EE (Dandi et al., 2018; Doreste-Mendez et al., 2019; Koe et al., 2016; McKibben and Dwivedi, 2021a; Cevik et al., 2023). Other studies, on the contrary, report MS-induced elevated CORT concentrations and a rescuing (Francis et al., 2002; Joushi et al., 2021) and preventive EE effect (Hegde et al., 2020). Furthermore, a study reported that the CORT response of MS animals to an acute stressor was greater than the respective CTL group, which was normalized by EE (Dandi et al., 2018). This result highlights the importance of evaluating not only basal CORT concentrations, but the magnitude of responses to acute stressors.

However, one study reported that MS induces higher CORT levels, without reversal by EE (McKibben and Dwivedi, 2021a), and another study showed that, despite no impact of MS on CORT concentration, EE increased the levels in previously MS-exposed animals (Odeon and Acosta, 2019). EE is considered a mild or moderate chronic stressor, increasing CORT levels. Thus, despite the conflict between the results of different studies on this parameter, animals housed in enriched environments may display greater CORT concentrations than animals housed in standard cages, which does not necessarily indicate negative effects, but rather greater adaptation and resilience to stress via stress inoculation, resulting from the amount of stimuli and constant novelty (Fox et al., 2006; Crofton et al., 2015; Macartney et al., 2022).

Similar to the age-dependent effect of EE on these emotional behaviors, EE protocols performed in the prepubertal and pubertal phases reverse the increase in CORT concentration induced by MS (Ji et al., 2022a; Huang et al., 2021). Regarding the effect of EE in adulthood, the results are more variable, indicating no effect (Ji et al., 2022b) or decreased CORT concentrations in both the MS and CTL groups (Huang et al., 2021).

The expression of GR is an important parameter to evaluate the activity of the HPA axis. EE augmented GR expression in the CA1 and DG areas of the hippocampus only in CTL animals, without any effect of MS (Vivinetto et al., 2013). In addition, EE did not reverse MS-induced lower GR expression in the hippocampus (Francis et al., 2002) or prevent it in the BLA (Hegde et al., 2020).

MS induces region-specific changes in the hippocampal CRH system. At the transcriptional level, MS upregulates CRH expression in CA1/CA2 regions. These changes translate to elevated CRH protein levels throughout most hippocampal regions (Francis et al., 2002; Wang et al., 2014; Wei et al., 2023), except for the DG where levels decrease. Furthermore, MS upregulates CRHR1 expression in CA3, and together these alterations in ligand and receptor expression promote heightened synaptic excitability. EE affects this system in a bidirectional way: when applied alone, EE elevates CRH levels in the CA1 and CRHR1 in the CA3, but when applied after MS it partially normalizes these alterations, restoring CRH/CRHR1 levels toward control values. Thus, both MS and EE shape the CRH/CRHR1 system in a region-specific manner, with MS creating a stress-sensitized phenotype and EE exerting either exacerbating or reparative effects, depending on prior experience (Wei et al., 2023). In the lateral habenula (LHb), CRH serves as a local modulator of excitability and synaptic plasticity, contributing to anxiety- and depression-like states. MS reduces CRH levels in this region, whereas EE has the opposite effect, which is associated with increased anxiety and depressive behavior in females. These findings suggest that EE can paradoxically amplify LHb responsiveness to aversive stimuli, depending on the animal's early-life history and the local dynamics of CRH signaling (Wei et al., 2022).

MS also induces epigenetic alterations on the *Crh* gene, increasing gene transcription by histone H3 hyperacetylation, DNA hypomethylation, and enhancing phosphorylation of the *Crh* gene promoter. The resulting persistent elevation of hippocampal CRH increases synaptic excitability and leads to impairments in hippocampal-dependent learning and memory. EE, in turn, reverses all of these neurobiological and behavioral alterations (Wang et al., 2014).

The activity of the HPA axis is influenced by OT, which attenuates stress responses to chronic situations and shows anxiolytic effects, and by vasopressin, which has anxiogenic action (Buckley and Ramachandran, 1981; Sapolsky et al., 2000; McCarthy et al., 1996). Fecal dosages of CORT, vasopressin and OT do not appear to be affected by the MS or EE protocols, only by the sex of the animals (Doreste-Mendez et al., 2019). On the other hand, quantification of OT in the central nervous system yields controversial results. MS reduces OT levels in the PVN and increases OTR expression in the hippocampus, particularly in CA1, thereby enhancing sensitivity to OT and disrupting the OT-CRH balance, which promotes a neural state more vulnerable to stress (Wei et al., 2023a, b). EE, in turn, reverses this oxytocinergic dysfunction by restoring OT levels (Wei et al., 2023), but modulates OTR in a region-specific manner, upregulating the receptor in CA3 while downregulating it in CA1 (Wei et al., 2023). OT and OTR also play a key role modulating emotional reactivity associated with the CRH system in the LHb. Similar to the hippocampus, MS decreases OT in the PVN while increasing OTR expression in the LHb, rendering this circuit more sensitive to oxytocinergic signaling and, consequently, more vulnerable to stress due to the disruption of the OT-CRH balance. In contrast, EE reverses these maladaptive changes by increasing systemic OT and reducing OTR expression in the LHb, restoring the OT-CRH axis and normalizing emotional responsiveness through OT interaction with CRH in the LHb (Wei et al., 2022).

### 3.6 Brain regions related to the behavioral effects

#### 3.6.1 Hippocampus and prefrontal cortex

Epidemiological studies demonstrate that individuals subjected to abuse during childhood present a significant reduction in hippocampal volume, which is associated with greater vulnerability to the development of depressive and post-traumatic stress disorders (Bremner et al., 1997, 2003; Vythilingam et al., 2002). Some evidence indicates a decrease in hippocampal volume in CA1 and CA3 in male Wistar rats submitted to 6 h of MS on PNDs 12, 14, 16, and 18 (Lehmann et al., 2002). However, 3 h of MS during the first two weeks of life had no effect on hippocampal volume in rats (Hui et al., 2010) or mice (Reshetnikov et al., 2021). Likewise, the results obtained to date on the effects of EE after MS on this parameter are also inconclusive. In one study, EE did not restore the reduction of total brain volume induced by MS (Irie et al., 2023), whereas another reported no effect of MS or EE on hippocampal volume (Hui et al., 2011). However, there are also results that EE increases prefrontal cortex (PFC) volume in both MS and CTL groups, that of the dorsal and ventral hippocampus in MS animals, although in the CTL group this increase was only observed in the dorsal hippocampus, with a significant reduction in the ventral hippocampus (González-Pardo et al., 2019a). In contrast, the volumes of CA1 and DG and the infralimbic PFC were greater in the MS than in the CTL group, and this effect was exacerbated by EE (Mansouri et al., 2021). These results point not only to the beneficial effects of EE, but also to its region-specific action in relation to the cerebral hemispheres. Furthermore, the opposite effects of EE on CTL and MS groups as to the ventral hippocampus suggest that there is not only a region specificity, but also a modulation by previous experiences (González-Pardo et al., 2019a).

Adolescent (Lajud et al., 2012), adult (Hulshof et al., 2011; Mirescu et al., 2004), and aged animals (Suri et al., 2013) exposed to 3 h of MS between PNDs 1 and 15 showed decreased cell proliferation and fewer immature neurons in the DG, although no effects on cell differentiation or neuronal survival have been reported (Hulshof et al., 2011). In contrast, other studies reported that MS increased hippocampal neurogenesis in peripubertal (Suri et al., 2013) and adult rats (Greisen et al., 2005). When MS impaired hippocampal neurogenesis, EE reversed (Hui et al., 2011) or partially restored deficits in DG maturation, morphology, synapses, and neuronal integration (Wei et al., 2023a, b). Adding to the inconsistency, some studies indicated no effects of MS or EE on hippocampal neurogenesis in mice (Navailles et al., 2008) or on PFC neurogenesis in rats (Koe et al., 2016; Irie et al., 2023). EE also increased nuclear density in the LHb (Wei et al., 2022), a region involved in emotional and cognitive regulation. MS decreased GABAergic parvalbumin interneurons in the PFC of male rats, with mixed findings regarding EE's ability to reverse this effect (do Prado et al., 2016; Irie et al., 2023). The loss of these interneurons was associated with cognitive deficits and was observed in schizophrenia patients (Lewis et al., 2005). Overall, while EE generally enhanced neurogenesis and MS tended to reduce it, variability in protocols and strains, combined with MS–EE interactions, led to complex and inconsistent outcomes that required further investigation.

Additional parameters of neuronal activity can be analyzed. LTP was significantly impaired by MS, indicating deficient hippocampal glutamatergic synaptic plasticity. In this case, this effect is reversed by EE, but also improved in the CTL group (Wang et al., 2014). On the other hand, MS decreases expression of hippocampal c-Fos, Grin2a and Grin2b genes, which are related to neuronal activation and excitatory neurotransmission, and EE does not reverse this effect (Cevik et al., 2023), although EE alone increases the expression of c-Fos in the hippocampus (Vivinetto et al., 2013). The sensitivity of some areas to environmental influence shows variation, since higher c-Fos expression in EE groups is found in the CA1, but not in the DG and the CA3 (Vivinetto et al., 2013). Furthermore, different housing conditions lead to recruitment of distinct brain areas to perform the same tasks and individual variability is more evident in EE animals (González-Pardo et al., 2019a).

Synaptophysin (SYP) and brain-derived neurotrophic factor (BDNF) are important markers of synaptic plasticity and both are reduced in the hippocampus and prefrontal cortex by MS (Aisa et al., 2009; Bachiller et al., 2020; Cui et al., 2020; Liu et al., 2018). In most studies included in this review, MS caused a reduction in the expression of BDNF receptors (Dandi et al., 2018; Menezes et al., 2020), gene expression (Cevik et al., 2023), plasma (Joushi et al., 2021), and hippocampal levels (Huang et al., 2021), and EE reversed most of these effects (Dandi et al., 2018; Menezes et al., 2020; Joushi et al., 2021; Huang et al., 2021), as well as increases SYP expression (Dandi et al., 2018; Wei et al., 2023). However, MS effects on BDNF signaling remain inconsistent, with studies showing no change in hippocampal BDNF receptors (Cordier et al., 2021) or increased plasma BDNF (Mansouri et al., 2021). Yet, both studies found that EE consistently upregulates BDNF expression regardless of MS effects.

In this case, sex and the period of life in which the EE protocol is performed also influence the results. EE from weaning until adulthood does not change SYP expression, however, when employed during adulthood, EE increases SYN expression in male, but not in female rats (Doreste-Mendez et al., 2019). For BDNF, the results point in the opposite direction. EE from weaning to late adolescence has a more robust effect in both MS and CTL groups than EE during adulthood (Huang et al., 2021). Furthermore, MS down-regulates PI3K-AKT signaling, one of the main signal transduction pathways of BDNF, which mediates cell survival, migration, angiogenesis, and neuroprotective effects. Similarly, EE reverses this adverse effect with greater efficiency in juvenility and adolescence (Huang et al., 2021).

#### 3.6.2 Basolateral amygdala

The BLA is related to the generation and maintenance of conditioned fear and generalized anxiety, characteristics of chronic stress in both humans and rodents (Davis et al., 2010). MS increases both neuronal activation and intrinsic neuronal excitability in the BLA. These changes enhance the excitatory synaptic transmission onto BLA projection neurons, which is correlated to increased anxiety-like behavior (Qin et al., 2021). One paper shows that MS increases the neuronal number and dendritic length and density in the BLA, and this effect is reversed by EE (Koe et al., 2016).

In addition to the potential of EE to reverse the deleterious effects of MS at a behavioral and neurobiological level, the protocol can also prevent future impairments. Hegde and colleagues (2020) demonstrated that MS increases neurogenesis in BLA, but EE employed between the PNDs 2 and 21, prevents this structural modification, which is associated with the decreased expression of anxiety-like behavior on OF (Hegde et al., 2020).

## 4 Discussion

**Fig. 5:**
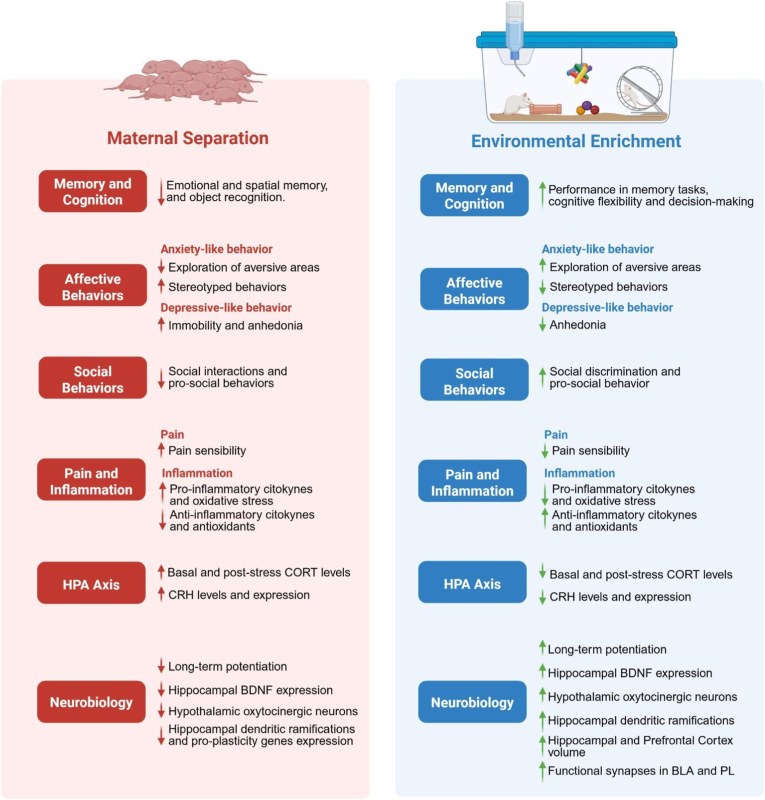
Graphical abstract of the main results presented in this review. BDNF: brain-derived neurotrophic factor; CORT: corticosterone; CRH: corticotropin-releasing factor; BLA: basolateral amygdala; PL: prelimbic cortex. Figure created in BioRender.com.

A summary of the main results described in this review is presented in Fig. 5.

### 4.1 Prevention and reversal

All studies included in this review sought to answer whether or not EE can be used as a strategy to reverse or mitigate the deleterious effects of MS, except for two that focused on the preventive effects of EE (Hegde et al., 2020; Ji and Xia, 2022), and both studies showed preventive effects of EE in the expression of anxious and depressive behaviors and pain sensitivity, in addition to the effects at the neuronal level and related to HPA axis activity.

Further studies are necessary to investigate the preventive nature of EE in different stress protocols in childhood to better elucidate the behavioral and neurobiological impacts. It is also important to clearly establish how EE during the neonatal period influences both the offspring's and the mother's wellbeing. Could EE improve the quality of maternal care and the mother-pup relationship? One study showed that this may be the case, with dams subjected to EE protocols before, during or after pregnancy taking better care of their pups and displaying less anxious- and depressive-like behaviors. Interestingly, the offspring of these EE mothers also display less affective behaviors in adulthood (Sparling et al., 2020).

### 4.2 EE-induced response variability and stress resilience

We observed a great variability in the results, likely due to differences in MS and EE protocols. Variations in MS protocols include the age of the litter and the duration of separation. Regarding EE, distinct cage sizes, the types of stimuli in the enriched cage (of physical and/or social), different environmental configurations, frequency of object changing and protocol duration are sources of variability. For instance, 11 of the 36 studies included in this review did not have the running wheel in the EE protocol. Both enrichment with objects and voluntary physical exercise significantly increased hippocampal neurogenesis, but through distinct neural pathways (Olson et al., 2006), indicating that differences in the protocol can alter the behavioral and neurobiological outcomes. As described above, the behavioral tests, species and strain used, the age of testing and the sex increases the variability of the results. Of the studies selected, 26 used rats, (19 studies used Wistar and 5, Sprague Dawley and 2, Holtzman rats), and only 10 used mice (5 studies with Balb/c, 4 with C57Bl and 1 with both strains). Despite the similarity of the behavioral results, the neurobiological differences between the two species are more evident.

Although sex is a biological variable (Shansky and Murphy, 2021) it is necessary to change the scientific culture to include both sexes and ascertain the generalizability or not of the results described. Less than half of the studies in this review (15 out of 36) included females. Evasion to include females is usually justified by variation in sex hormones that could interfere with the results. However, a meta-analysis showed that females are not more variable than males in almost all behavioral and neurobiological variables analyzed (Becker et al., 2016). Therefore, it is crucial to consider the representativeness and natural variability of responses between males and females (Becegato and Silva, 2024). One consistent sex difference that was clear from the analysis of the studies is that males respond faster to EE than females on several parameters (Réus et al., 2015; Ji et al., 2022a). These results reinforces the importance of including females in studies and developing experimental designs that aim to investigate these sexual differences, in order to improve the methods currently used in research, and developing personalized and appropriate treatments, increasing the translational power of the preclinical studies.

The neurodevelopmental period and the duration of EE protocols also need to be considered. Aversive experiences throughout life, especially in the early stages, can program the individual in a more or less adaptive way (Lupien et al., 2009; Oitzl et al., 2010; Schmidt, 2011; Teicher et al., 2003). EE in childhood and adolescence can act as an important modulatory factor during these neurodevelopmental windows. Thus, these critical periods should be seen not only as windows of vulnerability, but also as windows of opportunities to reprogram possible deleterious effects (Chelini et al., 2022). For this reason, most studies carry out EE protocols between the peripubertal and pubertal periods, i.e., from weaning to late adolescence or early adulthood. EE during the prepubertal and pubertal period reverses MS-induced non-adaptive responses more significantly compared to protocols in adulthood (Ji et al., 2022b; Huang et al., 2021), so it seems that the earlier EE is applied, the shorter the duration necessary for observing its beneficial effects. Many studies employ long EE protocols, lasting between 1 and 2 months. However, depending on the experimental design, shorter periods are effective and the beneficial effects persist for some time after the animals are transferred to standard housing (Francis et al., 2002; Koe et al., 2016; Dandi et al., 2018; González-Pardo et al., 2019a; Menezes et al., 2020). These findings reinforce the importance of early developmental stages for reprogramming of long-term adaptive responses and the building of resilience.

Individual variability within a strain and among strains further increases the analytical complexity specially with previous history of manipulations such as MS and EE. The outcomes most likely result from a combination of environmental and genetic factors (Plotsky et al., 1998) and, ideally, they should be considered and biases should be controlled as much as possible (Kempermann, 2019). When housed in an enriched cage, each animal interacts with the environment and the available objects in a particular way, according to its perception and interpretation of the stimuli present in the cage, leading to greater variability than that seen in animals housed in standard cages (Kempermann, 2019).

Standard cages provide an impoverished environment that limits the expression of natural behaviors. EE offers a more ethical and natural housing condition and can improve reproducibility when appropriately implemented (Loss et al., 2021). Although standard cage was adopted to reduce costs and variability, it fails to reflect animals’ natural variability, which is inherent to behavioral and neurobiological processes. This mismatch widens the gap between preclinical and clinical research, reducing translational value, as humans live in far more complex environments (Baumans and Van Loo, 2013; Baumans, 2010; Mo et al., 2016).

An important point to be highlighted is that most studies use EE as a means and not as an end, i.e., as the specific objective of the study. EE is normally used as a non-pharmacological intervention for prevention or reversal of the impairing effects of some stress protocols, but few original studies tried to compare the effects of different EE configurations in order to determine what are the essential elements that need to be included in the cage. As a consequence, the way EE is performed is extremely diverse among research groups, which contributes to the variability of results and difficulty of interpretation (Simpson and Kelly, 2011). Therefore, it is important to propose a standardization of EE protocols, based on intrinsic characteristics of the experimental animals and understanding the specific function of each item placed in the cage. Reducing variability of the protocols can increase consensus and help understanding the mechanisms mediating EE beneficial effects.

Finally, an increasingly strong hypothesis that explains the beneficial effects of EE on animals that have been previously subjected to some early life adversity is the stress inoculation or match-mismatch theory. While the cumulative stress hypothesis postulates that early stress increases vulnerability to future aversive experiences, the match-mismatch hypothesis postulates that exposure to mild or moderate stressors throughout life prepares the organism for future adversities and stressful experiences, developing resilience and more effective coping strategies, generating adaptive responses (Daskalakis et al., 2013; Nederhof and Schmidt, 2012). Both theories are based on the fact that childhood and adolescence represent windows of opportunity for possible phenotypic programming or reprogramming (Andersen, 2003; Gluckman et al., 2005). In this scenario, EE would act as a mild or moderate chronic stressor, since constant exposure to new environmental configurations and objects promotes constant activation of the HPA axis in these animals. Higher population density can also add new challenges to the environment in the face of greater social complexity. Thus, the stress inoculation hypothesis helps in understanding that EE, as a chronic stressor, adapts the individual against subsequent stressors, making them more resilient through increased neuronal plasticity (Crofton et al., 2015). This theory is reinforced by evidence showing that EE is more effective in animals subjected to stress protocols than in CTL animals, which indicates that the previous experiences play an important role in the impact of this manipulation (Fox et al., 2006). This hypothesis helps to understand why MS-EE animals have more adaptive characteristics in some of the studies included in this review (Vivinetto et al., 2013; Cordier et al., 2021; Hegde et al., 2020; Mansouri et al., 2021; González-Pardo et al., 2019a), since these animals have already experienced adversity and modulation in the neonatal period, and when faced with EE, a new potentially challenging experience in adolescence or adulthood, they may be better prepared to deal with this new scenario and, potentially, have more resilient responses. Apparently, different stress and EE protocols interact synergistically and the presence of both manipulations would improve the animals' performance in several skills and competencies (Fox et al., 2006; Macartney et al., 2022). However, other evidence indicates that the overlap of unpredictable chronic stress and EE can increase anxiety-like behavior due to stimulus overload (Taveiros Silva et al., 2025). Furthermore, EE itself can induce anxious and depressive-type behaviors, depending on the animal's strain used (de Brito et al., 2025). The evidence obtained to date is still very conflicting, not necessarily because it is contradictory, but mainly because most studies do not consider individual factors in the analysis, such as genetic background and its interaction with the environment, which also plays a fundamental role in the individual predisposition to stress vulnerability or resilience.

## 5 Future perspectives: epigenetics and transgenerational effects and clinical research

In addition to the effects of EE on preventing or reversing MS effects, the evidence accumulated to date also provides other promising perspectives for this non-pharmacological strategy that may have a positive impact on neurodevelopment. In animals exposed to prenatal stress, EE prevents anxiety-like behavior, drug abuse, social deficits, and memory tasks impairment (Morley-Fletcher et al., 2003; Razavinasab et al., 2022; Zhang et al., 2012), protects them from prolonged HPA axis response and increased CORT secretion (Morley-Fletcher et al., 2003), preserves SYN and GR expression in the hippocampus (Li et al., 2012) and improves neuroimmunological regulation (McCreary and Metz, 2016; McCreary et al., 2016). EE applied before breeding in fathers and prenatally in mothers significantly alters the offspring's developmental trajectories (Mychasiuk et al., 2012). During gestation or lactation, EE improves maternal care in different rodent strains and reduces anxious and depressive-type behaviors in both mother and offspring (Sparling et al., 2020). One study indicates that EE shapes the maternal behavior by decreasing the time spent by dams on the nest, maintaining the frequency of licking and grooming, which mimics a more naturalistic behavior (Connors et al., 2015). EE also prevents the transgenerational effect of parental trauma (Gapp et al., 2016) and can reverse epigenetic changes caused by childhood stress (Borba et al., 2021). Nevertheless, male offspring of EE fathers showed poor behavioural adaptive responses when housed under standard conditions, which was not observed in females, and a short period of EE during adulthood was sufficient to reverse these behavioural deficits (Hoffmann et al., 2020). Thus, just as stress can exert detrimental transgenerational effects, preconception and prenatal EE can enhance the quality of life of parents and their offspring, potentially improving resilience in the next generation (McCreary et al., 2016; Mychasiuk et al., 2012), particularly when EE conditions are maintained during development (Hoffmann et al., 2020). These effects are promoted by increased brain epigenetic plasticity later in life and the effects of prenatal experience on the adult brain epigenome seem to be more expressive than postnatal experiences (Mattern et al., 2019). Furthermore, EE therapeutic effects on social, anxiety-like, and depressive behaviors in female mice exposed to stress during gestation are similar to fluoxetine, and the combination of both treatments has a synergistic effect on these parameters (Bashiri et al., 2021).

In addition to the results observed in rodents, EE improved physical and psychological well-being in non-human primates, increasing species-typical behaviors, protecting against cognitive impairments, and decreasing maladaptive behaviors such as aggression, stereotyped behaviors, self-injury, anxiety and depression (Coleman and Novak, 2017; Lutz and Novak, 2005). Some clinical research with EE is underway. Cognitive and sensorimotor enrichment alleviates symptoms in children in the autistic spectrum (Woo and Leon, 2013; Woo et al., 2015), resulting in improvements in anxiety, sleep, communication, social and motor skills, sensory processing, communication, self-care, memory, and learning (Aronoff et al., 2016). Environmental stimulation based on educational enrichment and physical activity also reduces antisocial behavior in adolescents and schizotypal personality traits in children (Raine et al., 2003).

Epidemiological studies demonstrate the close relationship between chronic or high-intensity stress and the onset of emotional and mood disorders, with selective serotonin reuptake inhibitors (SSRIs) being the treatment of choice for children and adolescents (Bhatia and Bhatia, 2007). The SSRI improve symptoms of depression, anxiety (Bhatia and Bhatia, 2007; Caballero and Nahata, 2005; Whittington et al., 2004), cognitive and social functions (Teng et al., 2022). However, it also causes many adverse physiological and behavioral effects (Bridge et al., 2007; Mann et al., 2006; Murphy et al., 2021; Olfson et al., 2006). Thus, using drugs to treat psychiatric disorders in young people may represent a great risk, in the short- and long-term, due to the hormonal, neuronal, cognitive and morpho-functional changes during this developmental stage (Murphy et al., 2021). Moreover, social and environmental interactions play a crucial role in the development and progression of these disorders in children and adolescents (Bick and Nelson, 2016). These findings underscore the need to offer complementary therapies to enhance the effectiveness of traditional treatments to neuropsychiatric disorders that afflict children and adolescents. In this sense, EE, together with other non-pharmacological therapeutic strategies such as cognitive-behavioral therapy and physical exercise, can represent a promising perspective in promoting mental, psychological and emotional well-being in children and adolescents.

## 6 Conclusions

EE is a valuable tool to enhance physical and psychological well-being of laboratory animals, which is closer to natural living conditions, thereby producing results that may better reflect real-world scenarios than those obtained under standard housing, despite increased variability. Consequently, inconsistencies in the literature may stem from the wide diversity of both MS and EE protocols. Nevertheless, robust evidence indicates that EE is an effective non-pharmacological strategy able to reverse the negative effects of MS on memory and cognitive parameters, including its positive influence on neurogenesis. Regarding social, anxiety- and depression-like behaviors, as well as alcohol intake, findings remain heterogeneous, although they point to positive effects of EE. Therefore, this review highlights these beneficial effects and underscores the importance of including both males and females in experimental designs, as well as implementing EE protocols as early as possible during the neurodevelopmental period of laboratory animals.

## CRediT authorship contribution statement

**Natalia Ferreira de Sá:** Conceptualization, Data curation, Methodology, Writing – original draft, Writing – review & editing. **Nadyme Assad:** Resources, Writing – review & editing. **Rosana Camarini:** Conceptualization, Writing – original draft, Writing – review & editing. **Deborah Suchecki:** Conceptualization, Methodology, Supervision, Writing – original draft, Writing – review & editing.

## Declaration of competing interest

The authors declare no conflict of interest.

## Data Availability

No data was used for the research described in the article.
